# Disseminated tumour cells in bone marrow are the source of cancer relapse after therapy

**DOI:** 10.1111/jcmm.13867

**Published:** 2018-09-26

**Authors:** Buqing Sai, Juanjuan Xiang

**Affiliations:** ^1^ Hunan Cancer Hospital The Affiliated Cancer Hospital of Xiangya School of Medicine Central South University Changsha Hunan China; ^2^ Cancer Research Institute School of Basic Medical Science Central South University Changsha Hunan China; ^3^ The Key Laboratory of Carcinogenesis of the Chinese Ministry of Health Xiangya Hospital Central South University Changsha Hunan China; ^4^ Hunan Key Laboratory of Nonresolving Inflammation and Cancer Changsha Hunan China

**Keywords:** cancer relapse, circulating tumour cells, disseminated tumour cell

## Abstract

Accumulating evidence indicates that cancer cells spread much earlier than was previously believed. Recent technological advances have greatly improved the detection methods of circulating tumour cells (CTCs), suggesting that the dissemination of cancer cells into the circulation occurs randomly. Most CTCs die in circulation as a result of shear stress and/or anoikis. However, the persistence of disseminated tumour cells (DTCs) in the bone marrow is the result of interaction of DTCs with bone marrow microenvironment. DTCs in the bone marrow undergo successive clonal expansions and a parallel progression that leads to new variants. Compared to the CTCs, DTCs in the bone marrow have a unique signature, which displayed dormant, mesenchymal phenotype and osteoblast‐like or osteoclast‐like phenotype. The persistence of DTCs in the bone marrow is always related to minimal residual diseases (MRDs). This review outlines the difference between CTCs and DTCs in the bone marrow and describes how this difference affects the clinical values of CTCs and DTCs, such as metastasis and recurrence. We suggest that DTCs remaining in the bone marrow after therapy can be used as a superior marker in comparison with CTCs to define patients with an unfavourable prognosis and may therefore be a potential prognostic factor and therapeutic target for cancer therapy.

## 1 BACKGROUND

Metastasis is a major reason for the poor prognosis of patients with cancer and is responsible for over 90% of cancer‐related deaths.^1^, ^2^, ^3^, ^4^ Metastases occur when cancer cells dissociate from the primary cancer and enter into the circulation.^5^ Circulating tumour cells (CTCs) spread out through circulation and may subsequently reside in the permissive target tissues,^6^ in which case the cells are called disseminated tumour cells (DTCs). Disseminated tumour cells from various types of cancers are often found in specific organs, including bone marrow and lymph nodes.^1^, ^2^, ^7^ Research on the roles of CTCs and DTCs in bone marrow in the evaluation of cancer prognosis has grown exponentially. Significant evolution often occurs during cancer progression, generating variability between the primary cancer, CTCs and DTCs in the bone marrow. In this review, we summarize the difference between CTCs and DTCs and describe how this difference affects the clinical values of CTCs and DTCs, such as metastasis and recurrence. We suggest that DTCs in the bone marrow are the origin of cancer relapse and may therefore be a potential prognostic factor and therapeutic target for cancer therapy.

## 2 CANCER CELL DISSEMINATION IS AN EARLY EVENT

Cancer cell dissemination has long been considered to be a late event in tumour development. However, accumulating evidence indicates that cancer cells spread much earlier than was previously believed,^8^ even before the primary tumour is detected.^9^ Tumour cells are frequently detected in the blood and bone marrow of cancer patients who have no clinical or even histopathologic signs of metastasis.^10^ The variability in detection rates is likely due to differences in selection criteria and methodologies (Table 1). Recent technological advances have greatly improved CTC detection methods. An advanced unique microfluidic platform (CTC‐Chip) was found to identify CTCs in the peripheral blood of more than 90% of patients with metastatic lung, prostate, pancreatic, breast cancer and colon cancer and did not detect CTCs in the healthy control. In addition, CTCs were isolated in 100% of patients with early‐stage prostate cancer using the same platform,^11^, ^12^ indicating that the dissemination of cancer cells into the circulation may occur randomly. CTCs that home to the bone marrow are also detected in patients with pre‐invasive lesions, suggesting that bloodborne dissemination is also an early event.^12^ Given the much lower incidences of metastasis, the correlation between CTCs, DTCs and metastasis remains elusive. To date, the detection of CTCs and DTCs remains a challenging diagnostic approach and prognostic biomarker, not only as a result of methodological limitations but also because the heterogeneity among CTCs and DTCs in bone marrow compromises their ability to predict the metastatic behaviours. Neither CTC status nor DTC status has been included in routine clinical analysis.^13^

**Table 1:** Clinical relevance of different detection of CTCs or DTCs

Type	n	CTC/DTC	Measurement	Positive (%)	References
Gastric cancer	81	CTC	A45‐B/B3, vimentin, CD45	63	131
		Circulating tumour microemboli (CTM)		18.6	
Colon cancer	299	CTC	CK20,RT‐PCR	37.4	132
	227	DTC	CK20	35.7	
	61		BER‐EP4	19.7	
	134		A45‐B/B3	22.4	
Breast cancer	83	CTC	A45‐B/B3, CD45	52 (≥5 CTCs)	133
	83 (underwent therapy)			25 (≥5 CTCs)	
Breast cancer	431	CTC	A45‐B/B3	13	134
	414	DTC	A45‐B/B3	24	
Breast cancer	350	DTC	EMA	25	119
Various cancers	116	CTC	Microfluidic platform (the “CTC‐chip”)	99	11
Prostate cancer	7	CTC	Microfluidic platform (the “CTC‐chip”)	100	11

A45B/B3 detects cytokeratins 8,18,19; AE1 detects cytokeratins 10,14,15,16 and 19; AE3 detects cytokeratins 1,2,3,4,5,6,7 and 8; BER‐EP4 detects EpCAM; EMA detects epithelial membrane antigen; Microfluidic platform (CTC‐chip):antibody (EpCAM)‐coated microposts.

## 3 BONE MARROW IS A RESERVOIR OF DISSEMINATED TUMOUR CELLS

Bone marrow is a critical site of immune cell development and erythropoiesis. The bone marrow parenchyma includes hematopoietic stem cells and hematopoietic progenitor cells. The stroma, which is composed of stromal stem cells, extracellular matrix and several types of secreted cytokines, is highly vascular and enriched with numerous blood vessels and capillaries.^14^ Bone marrow is also a niche for mature plasma cells and memory T cells.^15^ Bone marrow displays structural and functional features resembling a secondary lymphoid organ, providing appropriate support for T cells. Accumulated evidence demonstrates that, in addition to the hematopoietic progenitor cells, bone marrow contains various immune cells, including regulatory T cells, conventional T cells, B cells, dendritic cells, natural killer T (NKT) cells, neutrophils, myeloid‐derived suppressor cells and mesenchymal stem cells.^14^

Bone marrow diseases, including leukaemia, lymphoma, multiple myeloma anaemia and other life‐threatening diseases, lead to an abnormality in the production of mature blood cells. Bone marrow is a preferred metastatic site for several solid tumours, such as breast cancer, lung cancer, prostate cancer and others.^16^ Bone marrow also represents a sanctuary site for DTCs derived from various additional types of epithelial tumours.^16^ The presence of DTCs in the bone marrow is associated with not only bone metastasis but also the development of distant tumours. DTCs in the bone marrow may re‐enter the vasculature and disseminate secondarily throughout the body.^17^, ^18^

Although the dissemination of cancer cells into the circulation and the bone marrow is an efficient process, cancer metastasis is an inefficient process. The persistence of DTCs in the bone marrow is always related to minimal residual diseases (MRDs). The mechanism underlying the process of DTCs localizing to and colonizing the bone marrow is complex, and the homing and survival of DTCs are more like a selective process. In one study, intracardiac injection of tumour cells was performed to study late stage of metastasis.^19^ These DTCs may transform into more aggressive variants and grow out to overt metastasis.^20^ Disseminated tumour cells were found to gather in the bone marrow and display unique gene expression, including the significant enrichment of genes known to regulate interleukin‐6 (IL‐6) signalling, cell adhesion and angiogenesis. Bone marrow contains unique anatomic regions defined by specialized endothelium. The specialized vasculature expresses the adhesion molecule E‐selection and the chemoattractant SDF‐1.^21^ Silencing CXCR4, VLA4 and FAK can effectively decrease the homing phenomenon.^19^ Disseminated hormone receptor–positive breast cancer cells hunt for specific blood vessels in bone marrow that contain the molecule E‐selectin. Due to the key molecules on their surface that bind to E‐selectin, the cancer cells enter the spongy tissue inside bones, often lying dormant for years.^22^

## 4 BONE MARROW FORMS A PREMETASTATIC NICHE FOR DISSEMINATED TUMOUR CELLS

Secondary cancer growths do not occur randomly.^23^ Stephen Paget's “seed and soil” hypothesis proposed that the homing and settlement of cancer cells depend on the fertile “soil” provided by a given microenvironment. Bone marrow is a major target organ for metastasis, providing a fertile “soil” for circulating tumour cells to settle and repopulate.^24^ The fertile “soil” is sometimes formed before the arrival of circulating tumour cells, in which case it is termed a “premetastatic niche.” Cells in this favourable microenvironment or “premetastatic niche” secrete many cytokines and chemokines, attracting primary tumour cells to the niche as well as supporting their subsequent colonization. The formation of the premetastatic niche is influenced by the primary cancer.^25^ Recent studies suggest that primary tumour cell–derived extracellular vesicles (EV) contribute to distant metastasis effectively. EV carried miR‐122 suppress glucose uptake of microenvironmental cells by downregulating the glycolytic enzyme pyruvate kinase.^26^ Tumour exosomal small nuclear RNAs remodel the lung metastatic niche by activating alveolar epithelial TLR3 to recruit neutrophil.^27^

The premetastatic niche is defined as a microenvironment that is highly proinflammatory, adhesive, vascular, proproliferative and chemotactic. Integrins on both tumour cells and the supporting stromal cells in the bone marrow, such as osteoclasts, inflammatory cells and bone marrow stromal cells, play key roles in enhancing the localization of cancer cells to the bone marrow.^28^ The interaction of integrins and their ligands allows for the mobilization and adhesion of cancer cells to the bone marrow stromal cells (αvβ3, α5β1, α4β1),^29^, ^30^, ^31^ osteoclasts (αvβ3),^32^, ^33^ platelets (αvβ3, α1β3),^34^ bone marrow hematopoietic progenitor cells (α4β1)^28^, ^35^ and endothelial cells (α1β1, α2β1).^36^ Chemokines such as SDF‐1,^37^, ^38^ S100A8 and S100A9,^39^ which are produced by bone marrow–derived cells (BMDCs) or primary cancer cells, elicit the accumulation of cancer cells, endothelial cells and macrophages in the bone marrow.^39^

Cancer‐induced stress elicits BMDCs, such as bone marrow–derived hematopoietic progenitor cells, and the mobilization of stromal stem cells from the bone marrow into the primary cancer microenvironment.^40^ Bone marrow–derived mesenchymal stem cells have the potential to develop into cancer‐associated fibroblasts (CAFs).^41^ Il‐6 and GM‐CSF were identified as the key factors released from CAFs that promote tumour‐associated macrophages infiltration in the premetastatic niche.^42^ Cancer‐educated BMDCs move back into the circulation and home to tumour‐specific premetastatic sites or reprogramme the microenvironment in distant sites by secreting chemokines^41^ or cytokines before the arrival of tumour cells.^37^, ^39^ Interestingly, a subpopulation of bone marrow–derived stromal cells (MSCs), which express endothelial and pericytic cells surface markers (CD31 and CD146), has been reported to reduce cancer cell homing to the bone marrow^43^. Bone marrow–derived CD11b^+^ Jagged2^+^ cells infiltrate primary tumours and accelerate cancer cell EMT. Moreover, circulating CD11b^+^ Jagged2^+^ cells offer an indicator for metastasis of colorectal cancer cells.^44^ Cancer cells preferentially home to the osteoblastic niche in bone marrow, competing with normal hematopoietic stem cells.^45^, ^46^

## 5 CHARACTERIZATION OF CTCS AND DTCS IN BONE MARROW

### 5.1 Intratumour genetic heterogeneity and parallel tumour evolution

CTCs are derived from primary tumour sites and secondarily disseminated from metastatic tumours. DTCs in various organs have a unique signature that reveals their origin.^12^ The adaption of CTCs occurs upon contact with the specific environment, indicating that DTCs undergo successive clonal expansions and a parallel progression that leads to new variants.^12^

**Figure 1:**
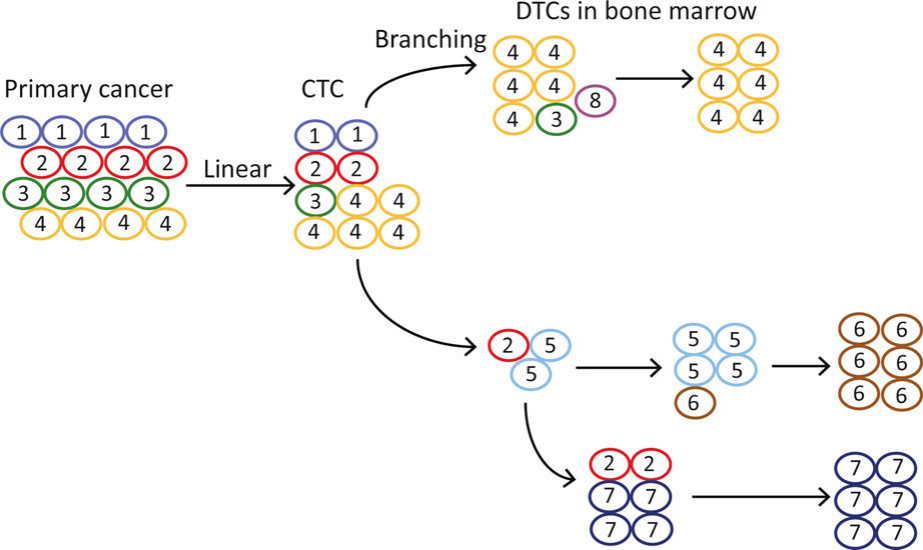
Linear and parallel clonal evolution in CTCs and DTCs in bone marrow. Left: Linear evolution. The final CTCs carry all mutations arising during the evolution. Right: Parallel evolution. CTCs circulated or arrived in bone marrow, the final DTCs in bone marrow may be dominated by a single done. Clones arise through divergent evolution. Numerals and circles colour indicate the subclones of the cancer cells

Next‐generation sequencing (NGS) and single‐cell sequencing have facilitated the detection of genome variation among CTCs and DTCs. Fluorescence in situ hybridization (FISH) analysis can be used to detect gene translocation.^47^ Multiregion genetic analysis of renal carcinomas revealed intratumour heterogeneity.^48^ The phylogenetic reconstruction of those renal carcinomas also revealed branched evolutionary tumour growth, with 63%‐69% of unique somatic mutations present in various tumour regions.^48^ Nonmutagenic treatments, such as everolimus, did not cause more mutations compared to pretreatment; on the contrary, certain mutations in the primary cancer disappeared after treatment with everolimus, indicating that the treatment resulted in clonal selection. Prostate cancer, which is histologically multifocal, is an example. The primary tumour foci from similar physical regions were found to be more closely related, suggesting that cancer arises from an ancestor and undergoes divergent evolution thereafter.^49^ The heterogeneity of CTCs reflects the heterogeneity of the primary cancer. Single‐cell deep sequencing studies have shown that mutations or somatic single nucleotide variants (sSNVs) in CTCs could be found in subclones of the primary tumour and metastases, indicating the cell origin of the CTCs.^49^ The identical genomic profiles for CTCs and the primary tumour reflect the linear model by which tumour cells are disseminated into the circulation. However, the colonization of CTCs in distant sites reflects the branching evolution model. CTCs overlap with more than 90% of primary cancer mutations but only 73% of metastatic mutations.^49^ When the DNA copy aberration of the cells (CNA) was compared, the DTCs in bone marrow showed 53% CNA similarity with that of the primary tumour, whereas the majority of the DTCs in the bone marrow displayed genomic profiles unrelated to the primary tumour, indicating that the parallel evolution model applies to the disseminated cells in the bone marrow (Figure 1).^50^, ^51^

### 5.2 Bone marrow is a dormant niche for DTCs

Metastasis is an inefficient process. Although the dissemination of cancer cells occurs at an early stage of cancer, many cancer cells delivered into the circulation either die or go into a dormant state.^52^ The following three types of gene expression signatures have been identified in CTCs: low‐proliferative signatures, biphenotypic signatures, in which both epithelial and mesenchymal markers are expressed, and epithelial‐stromal interface signatures, in which igfbp5 is expressed.^53^ Due to the short half‐life of CTCs in circulation, which persist for only 1‐2 hours, and due to apoptotic CTCs, the detection and prognostic value of CTCs in circulation has been unclear. Most CTCs die in circulation as a result of shear stress and/or anoikis.^54^, ^55^ Contact with integrins and exposure to bone‐derived cytokines in the bone marrow can reduce proapoptotic signalling.^56^, ^57^ The persistence of DTCs in the bone marrow during follow‐up is an indicator of the prognosis.^58^ Up to 40% of patients diagnosed with nonmetastatic breast cancer still have a significant risk of relapse, even after the complete surgical removal of the tumour, most likely because of the existence of DTCs.^51^, ^59^ DTCs in the bone marrow are usually considered to be nonproliferative and are believed to be the source of metastasis, independent of the primary tumour site and the pattern of overt metastases.^10^ More than 80% of patients with solid tumours harbour Ki67‐negative DTCs in the bone marrow.^60^ DTCs in the bone marrow exhibit very low or even no detectable pAKT levels.^61^ Cancer cells invade the bone marrow, where they remain dormant and are protected from chemotherapy or hormonal therapies that could otherwise eradicate them.

The quiescence of DTCs depends on the cross‐talk between the cancer cells and the bone marrow microenvironment. Bone marrow seems to be a particularly dormancy‐inducing environment for DTCs. Stable microvasculature constitutes a dormant niche for DTCs.^62^ Thrombospondin‐1 (TSP‐1) secretion by mature endothelial cells induces the sustained quiescence of breast cancer cells, whereas sprouting microvasculature secretes TGF‐β1 and periostin (POSTN) to promote tumour growth,^62^ indicating that the dormancy and re‐activation of DTCs are closely associated with the vascular basement membrane. Intravital microscopy images have shown that adhesion to the abluminal surface of the vasculature is one of the prerequisites for disseminated tumour cell survival.^63^ However, TGFβ2, which is present in bone marrow, induces dormancy through TGFβ receptor I, TGFβ receptor III and SMAD1/5 activation.^64^ Osteoclasts are specialized cells for the resorption of mineralized bone matrix. It has been reported that the proliferation of leukaemic cells is significantly suppressed when the cells are cocultured with osteoclasts.^65^ TGF‐β derived from osteoclasts was also shown to play roles in maintaining the quiescent state of cancer cells in the bone marrow.^65^

The HSC niche may act as a permissive site for DTCs to escape from source of stress. DTCs may reside in close proximity to osteoblasts while expressing high levels of Axl, one of the tyrosine kinase receptors for growth arrest–specific 6 (GAS6). The secretion of osteoblast‐derived GAS6 ligands by this niche can induce tumour cells to increase the ratio of GAS6 receptor Axl expression to Tyro3 expression which leads to a more dormant phenotype.^66^ DTCs recovered from the bone marrow are regulated by GAS6 through the MER/mTOR pathway, exhibiting a stem cell–like phenotype.^67^

### 5.3 Immuno‐mediated dormancy in bone marrow

The process of tumour immuno‐editing includes the following three key phases: elimination, equilibrium and escape.^68^ During the equilibrium phase, the immune system and the tumour cells experience mutual dynamic and constant selective pressure, and equilibrium between the immune response and the tumour cells results in a long‐term latency or relative dormancy.^69^, ^70^, ^71^ Cancer cells will resist the selective pressure from the immune system by acquiring mutations or undergoing other changes that allow for tumour progression in the face of an ongoing immune response.^72^, ^73^, ^74^ DTCs are characterized by a reduced expression of MHC class I molecules, which could result in these cells escaping the immune system.^75^, ^76^ Bone marrow aspirates from breast cancer patients with higher rates of overt bone metastasis more frequently revealed the absence of MHC I expression.^77^ Cell surface cytokeratin 8, 18, and 19 are trypsin‐sensitive factors that mask HLA class I molecules.^78^ FasL expressed on disseminated cancer cells mediates immune evasion by eliminating infiltrating lymphocytes.^79^, ^80^ Natural killer (NK) cells eliminate target cells with low or absent expression of MHC‐I. DTCs coated with platelets are conferred a false pretence phenotype, which helps them to evade NK cell–mediated cytotoxicity.^81^ In the interplay between DTCs and NK cells, the secretion of LDH5 and the ADAM10‑mediated shedding of the NKG2D ligand MICA/MICB from CTCs prevents the recognition and elimination of the cells via NK cell–mediated lysis.^82^, ^83^

On the other hand, cancer cells are regulated in the bone marrow by the immune system. The proportion of memory T cells among the CD4 and CD8 T cells was found to be much higher in the bone marrow of cancer patients compared with healthy donors (*P* < 0.001), suggesting that the immune equilibrium between DTCs and memory T cells is involved in the balance between dormant cancer cells and tumour escape.^84^ Only some of these immunological changes were also detectable in peripheral blood samples.^84^, ^85^ Functional immunosuppressive cells, such as MDSCs, Tregs and Bregs, are involved in the maintenance of DTC dormancy in the bone marrow.^86^, ^87^ This balanced state may be disturbed by both changes in the DTCs (eg, additional mutations or epigenetic modifications in genes controlling cell proliferation and apoptosis) and changes in the surrounding microenvironment (eg, the release of growth factors, angiogenic factors and cytokines).^17^, ^88^

### 5.4 DTCs in bone marrow showed mesenchymal phenotype

**Figure 2:**
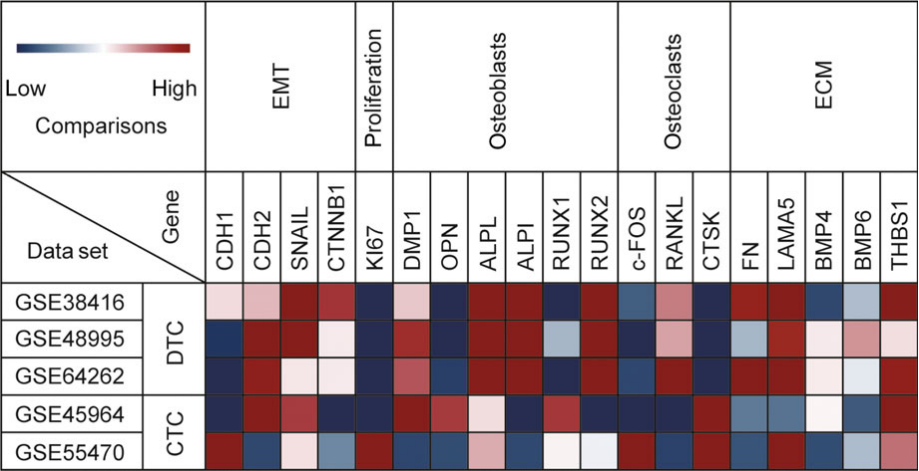
Heatmap representing expression profile of representative differentially expressed genes in DTCs and CTCs using Mev. The cDNA array data sets (GSE38416, GSE48995, GSE64262, GSE45964, GSE55470) were collected from the National Center for Biotechnology Information's Gene Expression Omnibus (GEO, NCBI)

Most DTCs in the bone marrow display properties of epithelial‐mesenchymal transition,^89^ during which they gain expression of the mesenchymal marker N‐cadherin and lose expression of the epithelial marker E‐cadherin (Figure 2).^90^, ^91^, ^92^, ^93^, ^94^ The synergistic effect of Snail and β‐catenin confers cancer cells the ability to survive during dissemination and invasion.^95^ Additionally, DTCs often display attributes of a stem cell–like phenotype (CD44^high^/CD24^low^) related to self‐renewal and angiogenesis.^96^, ^97^, ^98^, ^99^ This more plastic phenotype may be endowed by the primary tumour and clonally selected for during the process of homing, or promoted by the bone marrow niche as a stem cell niche to induce terminally differentiated cells to become stemlike.^46^ The mesenchymal phenotype may be a transient state of DTCs in the bone marrow, keeping them dormant and resistant to radiotherapy/chemotherapy.^100^ The overexpression of unfolded protein response (UPR) proteins and protein disulphide‐isomerase was observed in DTCs from bone marrow, suggesting that DTCs may resist the stress attributed to UPR phenotype.^96^

### 5.5 DTCs in the bone marrow displayed an osteoblast‐like or osteoclast‐like phenotype

Several distinct niches in the bone marrow, including the endosteal niche (osteoblasts) and the vascular niche, can protect DTCs from adjuvant therapies.^57^, ^101^ Transcriptome analyses of DTCs in the bone marrow have identified an osteoblast‐like or osteoclast‐like phenotype in which cancer cells in the bone marrow undergo an osteomimetism, expressing a pool of genes normally expressed by osteoblasts or osteoclasts.^12^ RANK‐L/RANK/OPG axis which regulates the process of bone turnover is activated in DTCs in bone marrow. Besides, the expression of genes participating in osteomimicry or osteolysis such as OPN, ALP and RUNX2 is also different from that of CTCs (Figure 2). Cancer cells have been shown to adopt an osteoblast‐like phenotype that may help them survive in the bone marrow.^102^ Cancer cells with an osteoblast‐like phenotype have been shown to exhibit an enhanced invasive ability.^103^ The dormant DTCs may be reactivated by the osteoclast‐mediated release of bone‐derived growth factors.^57^ The interaction between the bone marrow niche and the cancer cells was involved cancer‐derived E‐cadherin and osteogenic N‐cadherin.^104^

### 5.6 Human epidermal growth factor receptor 2 (HER2) and the estrogen receptor (ER)

Primary tumours and DTCs display a discordant ERα and HER2 status.^105^ This discordance may be important for determining which patients will benefit from endocrine and/or HER2 targeted therapy. Patients lacking ER or HER2 expression on the primary cancer but showing ER‐positive or HER2‐positive DTCs may benefit from an endocrine and/or HER2‐targeted therapy. This discordance may explain the failure rates seen in conventional endocrine adjuvant therapy for patients with DTCs that were ERα negative despite the presence of ERα positive primary tumours.^106^ Approximately 90% of analysed breast cancer patients with HER3 activation were found to exhibit very low or even no detectable levels of pAKT S473, suggesting that these cells might have fallen into dormancy.^61^

## 6 DTC IN BONE MARROW MAY BE A PROGNOSTIC BIOMARKER FOR RELAPSE AND A THERAPEUTIC TARGET FOR CANCER

Lymphatic metastasis has long been considered to be the primary route of metastasis, partly due to the accessibility of the lymph nodes. However, haematogenous metastasis is becoming increasingly recognized and confirmed because of the detection of CTCs. Lymph‐node involvement does not accurately predict the haematogenous dissemination of cancer cells, nor is haematogenous dissemination necessarily associated with lymph‐node involvement.^59^ Although the routine detection of CTCs has not been recommended as a prognostic method, numerous studies have demonstrated that the number of CTCs per mL is associated with the median overall survival.^107^ Hundreds of clinical trials incorporating CTC count as a biomarker in patients with various types of solid tumours are ongoing.^108^ CTCs thresholds range from 1 to 5 per mL for prognostic value in various cancer types, and the range for DTCs in the bone marrow is 1‐2 per 2 × 10^6^ cells.^109^ Sensitive immunocytochemical methods (ICCs), RT‐PCR, epithelial immunospot (EPISPOT), flow cytometry and a series of other strategies have overcome the difficulties in cell filtration, enrichment and identification. Equipment such as the FDA‐approved CellSearch^®^ system is already applied in tracking the CTCs in cancer patients.^110^ Clinically, liquid bioscopy helps to find DTCs in the bone marrow of patients with various types of solid tumours.^16^

CTCs and DTCs have been reported as independent prognostic markers that impact the progression‐free survival (PFS) and overall survival (OS).^13^ The number of CTCs is also correlated with serum levels of other markers, such as the prostate cancer biomarker, PSA.^49^ However, the inconsistency of these markers and the short half‐life of CTCs limit their application as diagnostic and prognostic biomarkers in the clinical setting. It has been considered that CTCs are singly suspended in the circulation, but the amount of CTC clusters can be underestimated due to the lack of appropriate detection methods.^111^ The survival rates of CTCs are highly variable, and EpCAM‐negative and CD44‐positive subsets exhibit quiescence properties and can initiate relapse or metastasis.^112^ The high heterogeneity of CTCs dilutes the gene signatures that are indicative of the phenotypic characteristics that allow CTCs to survive in distant organs. CTCs also include the DTCs in bone marrow that recirculate to other secondary organs or even back to the primary tumour site.^108^, ^113^ The detection of dormant DTCs enriched in the bone marrow may offer an ideal way to detect the minimal residue diseases. The detection rates at the same time in individual patients have always shown discordance of CTCs and DTCs, especially in patients after therapy.^114^ Some studies have compared the detection rates in the same patient. CTCs were detected in 10% of the patients, and DTCs were detected in 14% of the same patients.^115^ As DTCs in the bone marrow display a more malignant transformed phenotype, and the colonization of CTCs in the bone marrow is more like a selective process, DTCs in bone marrow are expected to be a superior marker for predicting overall survival, compared to CTCs. However,this conclusion is controversial.^114^, ^115^, ^116^, ^117^ Some studies have demonstrated a superior performance of DTCs in predicting overall survival in patients with cancer.^118^ In contrast, other studies reported that DTCs were associated with bone metastasis (*P* = 0.0001) but not with a poorer overall survival.^116^ At this time, DTCs have not been recommended as a substitute for CTCs as the prognostic biomarker, because bone marrow aspiration is invasive and uncomfortable for patients.

However, the presence of bone marrow DTCs in patients with primary breast cancer is associated with a shorter relapse‐free survival and locoregional relapse, although it is not an independent prognostic factor.^58^, ^119^ The persistence of DTCs during follow‐up has been shown to significantly predict the increased risk for subsequent relapse and death.^120^ The importance of histologic or genetic subclassification of cancer lies principally in the tumour biology, especially the response to therapy and the outcome. DTC status has been included in the new American Joint Committee on cancer classification.^13^ The presence of DTCs identified (at median 85 months follow‐up) a subgroup of luminal A patients characterized by high expression levels of ER‐related genes and low expression of the HER2 cluster and proliferation‐associated genes. This subgroup was found to have particularly poor outcomes (*P *=* *0.008); however, this finding was not apparent for other tumour subtypes.^121^ DTCs, but not CTCs, are associated with DFS in node‐negative patients.^115^ Currently, no diagnostic tools are available to monitor the response after the completion of adjuvant treatment to identify patients who need secondary adjuvant therapy due to persistent tumour cell load. The persistence of DTCs in the bone marrow after a disease‐free follow‐up interval indicates that DTCs in the bone marrow may be used as a surrogate marker to predict the risk of relapse, especially to identify patients with a poor response to therapy. The reevaluation of bone marrow status may be a promising tool because the presence of DTCs is a possible surrogate marker for persistent MRD. Studies have shown that the persistence of DTCs in the bone marrow of patients with primary breast cancer after conventional adjuvant therapy is associated with a poor prognosis.^106^ The existence of dormant DTCs in the bone marrow increases the risk of late relapse. When patients were followed for years, DTCs in the bone marrow were found to be a superior marker for predicting relapse. The persistence of DTCs in the bone marrow during follow‐up significantly predicted the increased risk for subsequent relapse and could be used as an indicator for secondary treatment intervention.^120^ Combining the data from various laboratories, it is estimated that before treatment, the positivity of DTCs in the bone marrow is approximately 30%.^122^, ^123^ Chemotherapy or bisphosphonates can reduce the positivity of DTCs.^123^ Six months after treatment, DTCs remaining in the bone marrow after therapy define patients with an unfavourable prognosis.^122^ Several studies have connected the presence of occult metastases in the bone marrow with a higher risk of recurrence.^124^, ^125^ DTC status can be used to identify high‐risk patients after chemotherapy and guide treatment decisions. However, DTC status after surgery was not associated with overall survival (Table 2).^126^

**Table 2:** Prognostic relevance of DTCs or CTCs

n	DTC/CTC	Marker	Treatment	OS (pre)	OS (post)	DFS (pre)	DFS (post)	References
83	CTC	A45B/B3	ACT	0.0048	0.0029	0.0014	0.007	133
213	CTC	EpCAM, CK	NACT	0.0057(CTC≥1)	ns	0.031(CTC≥1)	0.43(CTC≥1)	135
				<0.0001(CTC≥2)	ns	<0.0001(CTC≥2)	0.69(CTC≥2)	
394	DTC	A45B/B3	ACT	0.156				136
47	DTC	A45B/B3	ACT	0.009		0.004		137
236	DTC	AE1/E3	NACT	0.671	<0.001			138
	CTC		NACT		0.318			
	DTC		Surgery	0.715				
	CTC		Surgery	0.631				
211	DTC		NACT			0.602	0.003	
	CTC		NACT			0.146	0.434	
	DTC		Surgery			0.48		
	CTC		Surgery			0.551		
1489	CTC	EpCAM	ACT	0.023	0.154	<0.0001	0.054	139
100	DTC	AE1/E3	ACT	<0.0001				140
129					0.92			
60	DTC	AE1/E3	Radiotherapy			ns	0.02	141
193	DTC	A45B/B3	NACT				0.0035	142
60	CTC	pan‐CK	ACT	0.002		<0.001		143
	DTC			0.0005		0.003		
103	DTC	CK20	NACT		0.04		0.03	144
117	CTC				ns		ns	

A45B/B3 detects cytokeratins 8,18,19; AE1 detects cytokeratins 10,14,15,16 and 19; AE3 detects cytokeratins 1,2,3,4,5,6,7 and 8; pre: DTC/CTC detection performed before treatment; post: DTC/CTC detection performed after treatment; ACT: adjuvant therapy; NACT: neoadjuvant therapy.

Analysis of bone marrow DTCs offers the possibility of developing targeted therapies to eliminate the residue of dormant cells.^127^ The eradication of DTCs by bisphosphonates has already been demonstrated, and it was recently demonstrated that clodronate is effective in DTC elimination even after years of first diagnosis.^13^ Treatment with clodronate has made a significant improvement in OS and significantly reduced the incidence of bone metastasis.^128^ Preventing dormant DTCs in the bone marrow from reactivating cell cycling or kicking dormant cancer cells back into circulation may be strategies for preventing the relapse of metastatic disease. β‐adrenergic receptor antagonists, which interfere with proliferative re‐activation norepinephrine (NE) signalling, may reduce cancer relapse or slow disease progression.^129^ One potential strategy is finding a way to inhibit E‐selectin, which could limit the ability of the cancer cells to travel into the bone and resurge as metastatic cancer. A combination of the E‐selectin inhibitor GMI‐1271, daunorubicin and cytarabine, was shown to result in a greater depletion of AML from the bone marrow.^130^ Moreover, GMI‐1271 was reported to enhance the response to chemotherapy. Bone marrow transplantation could also be a good choice after the surgery or chemotherapy.

## 7 CONCLUSION

Dissemination of cancer cells is considered to be an early and random event in the process of cancer progression as detection methods have been greatly improved. DTCs in the bone marrow may be endowed with particular characteristics that are different from CTCs in the circulation by the special environment. These dormant, mesenchymal, osteoblast‐like or osteoclast‐like signatures may provide superior markers to CTCs for predicting metastasis or relapse of cancer and provide potential therapeutic targets for therapy.

## CONFLICTS OF INTEREST

The authors declare that they have no conflict of interests.
